# The Microbial Metagenome of Eluates Obtained From the Surface of Broccoli Heads Subjected to Different Light Treatments

**DOI:** 10.3389/fmicb.2022.820419

**Published:** 2022-04-13

**Authors:** Shixian Zeng, Jingchun Cui, Jinliang Xiong, Shuzhi Yuan, Xiaozhen Yue, Wenqiang Guan, Lipu Gao, Jia Liu, Jinhua Zuo, Qing Wang

**Affiliations:** ^1^Key Laboratory of the Vegetable Postharvest Treatment of Ministry of Agriculture, Beijing Key Laboratory of Fruits and Vegetable Storage and Processing, Institute of Agri-Food Processing and Nutrition (IAPN), Beijing Academy of Agriculture and Forestry Sciences, Beijing, China; ^2^Key Laboratory of Biology and Genetic Improvement of Horticultural Crops (North China) of Ministry of Agriculture, Key Laboratory of Urban Agriculture (North) of Ministry of Agriculture, National Engineering Research Center for Vegetables, Beijing Vegetable Research Center, Beijing Academy of Agriculture and Forestry Sciences, Beijing, China; ^3^College of Life Sciences, Dalian Minzu University, Dalian, China; ^4^Tianjin Key Laboratory of Food Biotechnology, Tianjin University of Commerce, Tianjin, China; ^5^Chongqing Key Laboratory of Economic Plant Biotechnology, College of Landscape Architecture and Life Science/Institute of Special Plants, Chongqing University of Arts and Sciences, Yongchuan, China

**Keywords:** broccoli, microbial diversity, foodborne pathogens, high-throughput sequencing, postharvest pathogens

## Abstract

Foodborne illnesses present a major threat to public health and are frequently attributed to foodborne pathogens present on fresh produce. Some opportunistic pathogens of broccoli are also responsible for causing head rot. Three different light treatments, UV-C, red LED (50 μml/m^2^/s), and UV-C + LED were used to treat broccoli prior to or during storage. Following the light treatments, microorganisms present in eluates obtained from the surface of broccoli heads were characterized using a metagenomic approach. Metagenomic DNA libraries were subjected to high-throughput sequencing on an Illumina Hiseq platform. Results indicated that the combined treatment of LED red light and UV-C provided the best sensory preservation of broccoli, followed by LED red light and then UV-C. The bacterial communities in the eluates obtained from the surface of broccoli heads in all three light treatments were primarily represented at the phylum level by *Proteobacteria* and *Firmicutes*, while fungal communities were primarily represented by *Ascomycota* and *Basidiomycota*. Further analysis indicated that the all three light treatments reduced the presence of foodborne pathogens and bacterial taxa responsible for broccoli spoilage. While UV-C had a significant inhibitory effect on *Botrytis cinerea*, the light treatments increased the relative abundance of *Pseudomonas fluorescens*. Results indicate that a metagenomic approach can be used to detect pathogenic bacteria and fungi on fresh vegetables and assess the impact of management practices, such as light treatments, designed to maintain postharvest quality, on the composition of the microbiome present on the surface of harvested produce.

## Introduction

Broccoli (*Brassica oleracea* var. Italica) is rich in ascorbic acid, glucosides, flavonoids, carotenoids, vitamins, and dietary fiber (Abdelfattah et al., 2016). It also possesses antioxidant and anti-cancer properties. In addition to the high content of antioxidants, such as ascorbic acid, polyphenols, and glucosinolates, broccoli also contains polyphenols. While broccoli contains a large number of gluconates with low antioxidant activity, their hydrolysates are considered cancer preventative nutraceuticals (Podsędek, 2007). Fresh broccoli, however, deteriorates and ages rapidly after harvest when stored at room temperature and is thus highly perishable. During the process of senescence, florets turn yellow and nutrient levels decrease rapidly (Nishikawa et al., 2003).

Bacteria and fungi inhabit the surface of fresh vegetables as they do the surface of other plant organs. Consumers are directly exposed to these microorganisms, which may have adverse health effects. Exposure to some bacteria may cause an allergic reaction in the respiratory tract of some people and/or affect the symbiotic flora inhabiting the gastrointestinal tract (Hanski et al., 2012). In fact, fresh vegetables have been reported to be a major source of microorganisms and microbial proliferation in indoor environments (Flores et al., 2013). Bacteria living on the surface of plants can play a positive or negative role when they are ingested. For example, foodborne pathogens can cause severe illness and death when ingested and are responsible for major outbreaks of foodborne diseases (Dong et al., 2020). The interactions that occur between plants and their resident microorganisms are critical to plant health (Cordovez et al., 2019). Kim and Park (2021) reported that the fungal community plays an important role in the postharvest microbiome of broccoli florets, the latter of which can have a large potential impact on the spoilage of stored produce. In this regard, a thorough understanding of the microbial ecology of harvested vegetables can be used to improve the safety and quality of fresh vegetables.

A variety of safety and quality-preserving measures have been developed to reduce the contamination of harvested vegetables with foodborne pathogens and the proliferation of resident microorganisms in general (Toivonen, 2009). Sanitation with chlorine solutions is typically used in commercial settings to reduce microbial loads on harvested fruits and vegetables, however, the formation of chlorine halogenated side products can also have a negative impact on human health and the environment.

Light is a major factor affecting the physiology of plants and specific wavelengths have been used to prolong the shelf life of fresh-cut broccoli by 3 days and also maintain its nutritional quality (Zhan et al., 2012). UV light has been used for decades for water treatment, as well as surface and air disinfection. In particular, UV-C (wavelength of 220–300 nm) light has been shown to extend the shelf life of whole and fresh-cut fruits and vegetables. The United States Food and Drug Administration (FDA) has approved the use of UV irradiation to control pathogens in unprocessed and processed food (USFDA, 2000). Sufficient exposure to UV is lethal to most microorganisms, including those that cause fruit and vegetable spoilage. UV irradiation is a non-thermal, non-ionizing, treatment technology, which has little effect on heat-sensitive nutrients or the general chemical properties of products. It does not leave any residues when it is used as a disinfectant and, thus, represents an environmentally friendly method of sterilization (Sethi et al., 2018). The use of UV light emitting diodes (LED) is an effective and economical way to treat vegetables as LED lights are small, safe, stable, and emit cold light (Morrow, 2008). The use of a variety of LED treatments has been reported to maintain the postharvest quality of vegetables. For example, red LED light irradiation was effective in delaying broccoli senescence after harvest (Ma et al., 2014). White LED light irradiation was reported to elevate vitamin C levels in Chinese kale sprouts, and in the same study blue LED light irradiation significantly enhanced the level of total phenolics, anthocyanins, and antioxidant capacity (Qian et al., 2016).

Culture methods have been traditionally used to isolate, grow, and identify pathogens, and microorganisms in general, but is time-consuming and laborious, especially when attempts are made to identify a large number of taxa (Chen et al., 2010). More recently, metagenomic sequencing, employing high-throughput sequencing technologies, has been used to assess microbial diversity and overcome the limitations of the culture method (Qin et al., 2010). Therefore, metagenomic sequencing was used in the present study to assess and identify the microbial community present on broccoli exposed to UV-C and LED light treatments and stored for 3 d.

## Materials and Methods

### Sample Collection

Commercially mature broccoli heads were collected on August 13, 2019 from Guyuan, Hebei Province, China and immediately transported to the laboratory that day. Broccoli heads that were uniform in size, shape, color, and without physical or disease injury were selected for use in the study. The selected broccoli heads were randomly divided into four groups, with three biological replicates in each group and five broccoli heads serving as a single replicate. The specifications for the UV-C, LED, UV-C + LED treatments were based on previous studies (Liu et al., 2020). There were four treatment groups: (1) Dark: broccoli heads were stored in the dark at 20°C, (2) UV-C: broccoli heads were irradiated with UV-C (3KJ/cm^2^) for 2 min and 29 s and then stored at 20°C in the dark, (3) LED: broccoli heads were stored at 20°C under red LED light (50 μml/m^2^/s), and (4) UL (UV-C + LED): broccoli heads were irradiated with UV-C (3KJ/cm^2^) for 2 min and 29 s and then stored at 20°C under red LED light. A DAT-TAL 115/230V UV radiometer (Cole-PARMER) was used to measure the amount of UV-C radiation administered. Broccoli heads were packed in polyethylene film (0.04 mm) bags prior to storage. Starting from day 0, samples were taken every day for a total of 3 days and the experiment was repeated three times. At the time of sampling, each broccoli head was sprayed with 25 ml phosphate buffer solution (0.1 M PBS) and the rinse solution was collected in a fresh-keeping bag sterilized with sodium hypochlorite and dried. The eluate was then placed in a 50-ml test tube and centrifuged at 12,000 × *g* at 4°C for 5–10 min. The supernatants were discarded and the precipitates were stored at −80°C.

### DNA Extraction

DNA was extracted from the precipitates obtained from the broccoli eluents using a PowerSoil DNA Isolation Kit (MoBio Laboratories, Carlsbad, CA) following the manufacturer’s directions. The purity and quality of the genomic DNA were checked on a 1% agarose gel.

### High-Throughput Sequencing

DNA was sheared to 300 bp with a Covaris ultrasonic crusher. The fragments were then end repaired, a poly A tail added, and then ligated with Illumina compatible adapters with indexes to prepare the sequencing libraries. The constructed DNA libraries were deep sequenced on an Illumina Hiseq platform at Allwegene Company (Beijing). Base calling and error estimation of the sequencing reads were performed using Illumina Analysis Pipeline Version 2.6 software.

### Data Analysis

Quality control of the raw data was carried out using Trimmomatic (Bolger et al., 2014) software, and included removal of the index and adapter sequence and low-quality reads. Reads were discarded if they if they contained adapter sequence, had an N (uncertain base) ratio greater than 1%, and if the low-quality base (Q ≤ 20) score was greater than 50%. Reads that were less than 150 bp were also discarded.

High-quality sequences were blasted against the NR database and classified into different taxonomic groups using the DIAMOND software program (Buchfink et al., 2015). MEGAHIT (Li et al., 2015) was used to assemble the sequences, and contigs <500 bp were filtered out. Contigs were annotated with Prodigal software (Hyatt et al., 2012) to predict open reading frames (ORFs), and CD-HIT software (Li et al., 2001) was used to construct the non-redundant gene set. Bowtie (Langmead et al., 2009) was used to compare the sequencing data with the non-redundant gene set, and the gene abundance in different samples was calculated.

## Results

### Effect of Different Light Treatments on the Sensory Attributes of Broccoli

**Figure 1:**
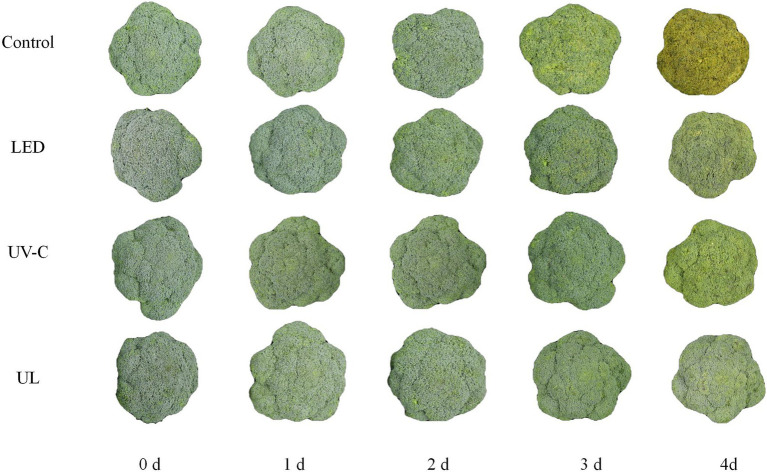
Broccoli treated with UV-C, LED red light and UL were stored at 20°C for 4 days.

After the treated broccoli was stored at 20°C for 3 days, it was observed that broccoli in the UV-C + red LED light treatment group had the freshest appearance, followed by red LED light and UV-C treatment groups. After 3 days of storage at 20°C, broccoli in the control group turned yellow and began to rot, while broccoli in the other 3 treatments did not turn yellow, and only a slight level of rot was present in the red LED and UL (UV + red LED light) treatment groups. On day 3 of storage, the broccoli in the control group had completely turned yellowed and decayed tissues were clearly evident. In contrast, broccoli in the UV-C group and LED group had a slightly yellow appearance and only slight evidence of decay, while the broccoli in the UL group had not yellowed and only exhibited slight evidence of decay (Figure 1).

### PLS-DA Analysis of Species Abundance in Eluents of Broccoli That Had Been Subjected to the Different Light Treatments

**Figure 2:**
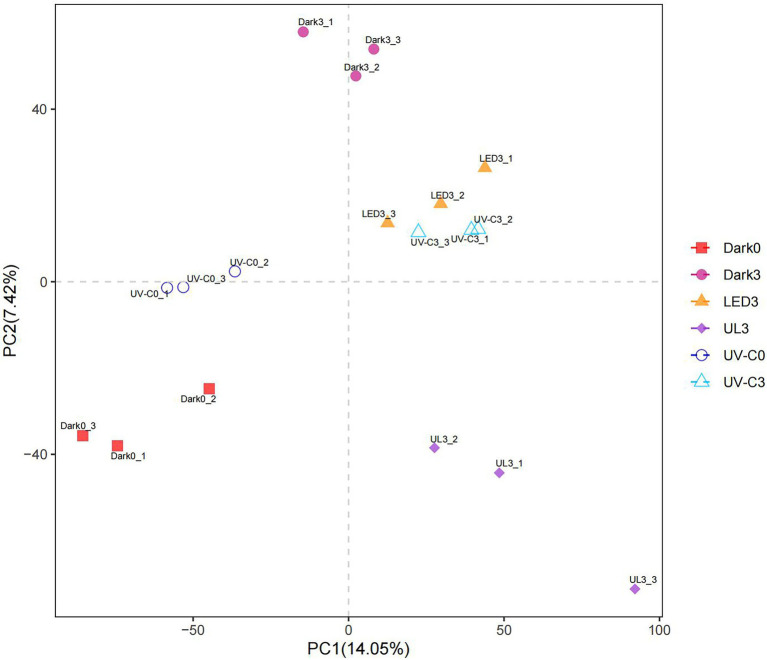
PLS-DA plot illustrating the effect of different light treatments on the relative abundance of microorganisms present on broccoli placed in storage for 3 days at 20°C. Dark0: initial value. The three replicates are Dark0_1, Dark0_2, and Dark0_3. Dark3 broccoli heads were stored in the dark for 3 days at 20°C. The three replicates are Dark3_1, Dark3_2, and Dark3_3. UV-C0: broccoli heads were irradiated with UV-C (3KJ/cm^2^) for 2 min and 29 s then sampled. The three replicates are UV-C0_1, UV-C0_2, and UV-C0_3. UV-C3: broccoli heads were irradiated with UV-C (3KJ/cm^2^) for 2 min and 29 s then stored at 20°C in the dark for 3 days. The three replicates are UV-C3_1, UV-C3_2, and UV-C3_3. LED3: broccoli heads were stored at 20°C under 50 μml/m^2^/s red LED light for 3 days. The three replicates are LED3_1, LED3_2, and LED3_3. UL (UV-C + LED): broccoli heads were irradiated with UV-C (3KJ/cm^2^) for 2 min and 29 s and then stored at 20°C under red LED light (50 μml/m^2^/s) for 3 days. The three replicates are UL3_1, UL3_2, and UL3_3.

Partial least squares-discriminant analysis (PLS-DA) is a PLS regression method with a special binary “dummy” y-variable and is commonly used for classification and biomarker selection in metabolomic studies (Szymańska et al., 2012). A PLS-DA analysis plot was constructed to visualize the relative similarities in species abundance between the different treatment groups (Figure 2). Results indicated that the species abundance in the treatment groups receiving irradiation was distinctly different from the untreated control.

### Effect of the Different Light Treatments on the Epiphytic Bacterial Community of Broccoli

**Figure 3:**
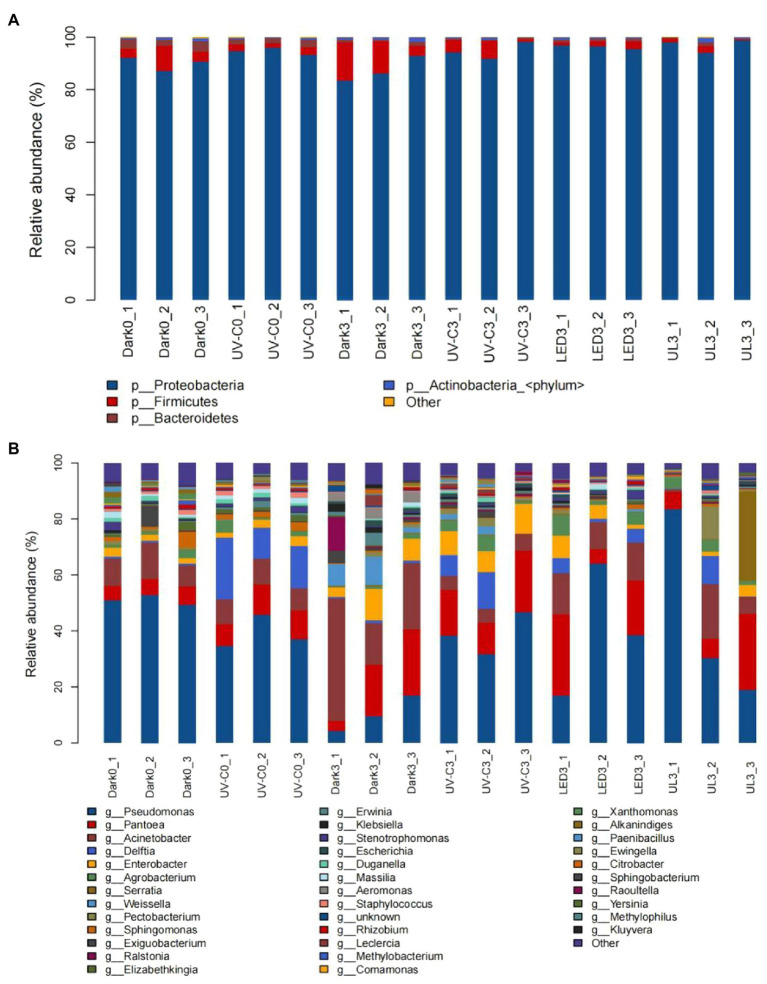
Taxonomic assignments of bacterial sequences at the level of phylum **(A)** and genus **(B)** obtained from eluates collected from the surface of broccoli subjected to different light treatments. Dark0: initial value. The three replicates are Dark0_1, Dark0_2, and Dark0_3. Dark3: broccoli heads were stored in the dark at 20°C for 3 days. The three replicates are Dark3_1, Dark3_2, and Dark3_3. UV-C0: broccoli heads were irradiated with UV-C (3KJ/cm^2^) for 2 min and 29 s then sampled. The three replicates are UV-C0_1, UV-C0_2, and UV-C0_3. UV-C3: broccoli heads were irradiated with UV-C (3KJ/cm^2^) for 2 min and 29 s then stored at 20°C in the dark for 3 days. The three replicates are UV-C3_1, UV-C3_2, and UV-C3_3. LED3: broccoli heads were stored at 20°C for 3 days under 50 μml/m^2^/s red LED light. The three replicates are LED3_1, LED3_2, and LED3_3. UL (UV-C + LED): broccoli heads were irradiated with UV-C (3KJ/cm^2^) for 2 min and 29 s and then stored at 20°C under red LED light (50 μml/m^2^/s) for 3 days. The three replicates are UL3_1, UL3_2, and UL3_3.

The relative abundance of epiphytic bacterial taxa in eluates collected from broccoli in the different light treatment groups is presented in Figure 3A. A total of 63 phyla, 156 orders, 379 families, and 1,568 genera were detected in the collective samples. At the phylum level, *Proteobacteria* accounted for 83.4%–98.7% of the total bacterial community. *Firmicutes* accounted for an additional 0.5%–14.6%. Other phyla, including the *Bacteroidetes* and *Actinobacteria*, collectively comprised an average of less than 5% of the total community. The bacterial taxa present in the eluates of broccoli that received different light treatments are presented in Figure 3B. *Pseudomonas* (4.4%–83.4%), *Pantoea* (3.4%–29%), *Acinetobacter* (8.4%–43.7%), and *Delftia* (0.1%–22.1%) were the dominant genera (Figure 3B).

Table 1 lists the relative abundance of the top 20 species found in the broccoli eluates. The relative abundance of these bacteria increased and decreased after 3 days of storage in the dark. Light treatment increased or maintained the relative abundance of most bacteria, relative to the control, and only inhibited a small number of bacteria, such as *Enterobacter cloacae complex*, *Acinetobacter bereziniae*, *Weissella cibaria*, *Acinetobacter baumannii*, *Acinetobacter johnsonii,* and *Pseudomonas protegens*.

**Table 1:** The relative abundance of the top 20 epiphytic bacterial species obtained from the surface of broccoli heads stored at 20°C for 3 days.

	Dark0	UV-C0	Dark3	UV-C3	LED3	UL3
*Pseudomonas fluorescens*	15.40417701	12.86211041	2.763489468	13.53619221	19.78216163	26.34984298
*Pantoea agglomerans*	3.764550987	7.478397613	10.86477311	12.01812651	13.64774288	10.19212068
*Delftia acidovorans*	0.546213802	12.48062223	0.561941167	5.479780247	2.961332048	2.672260671
*unidentified*	3.468184857	3.974605106	4.179155578	2.949569819	4.922277989	3.144516814
*Acinetobacter soli*	3.76767765	3.943325387	2.663842767	1.612350057	6.254581404	3.369530022
*Pseudomonas viridiflava*	4.973748462	6.110381421	1.239944766	5.019967977	2.393802582	1.196089213
*Pseudomonas syringae group*	0.314446211	0.692025467	0.085457749	0.345816391	0.138395472	0.106537126
*Enterobacter cloacae complex*	0.015780208	0.031002715	0.142638081	0.154557635	0.04348667	0.028828858
*Agrobacterium tumefaciens*	1.45070448	1.661477458	0.695439495	2.430496715	3.767025329	2.345769647
*Pseudomonas syringae group genomosp. 3*	2.381390979	3.316014177	0.855672113	2.372513764	1.740893483	0.935448793
*Acinetobacter beijerinckii*	2.214816777	1.289993749	2.893832568	1.409526544	1.85568993	1.243566413
*Serratia fonticola*	0.028374552	0.059360337	0.026857624	0.01724821	0.009303664	9.189040395
*Pseudomonas putida*	1.199608052	2.135331055	0.910773092	1.231904808	0.958906061	2.698105484
*Acinetobacter bereziniae*	0.028428709	0.044953599	8.65078502	0.047368271	0.129201026	0.039624465
*Weissella cibaria*	0.025519441	0.051115432	5.278651896	1.389922533	0.330766771	0.058962388
*Acinetobacter baumannii*	0.939176145	0.740448887	2.256356591	0.494815518	1.066196379	0.828463683
*Pantoea ananatis*	1.042809899	0.595914447	1.034214645	1.379536894	1.09348271	0.981452193
*Acinetobacter johnsonii*	0.258840868	0.462379457	3.876642591	0.315470884	0.148789585	0.981841603
*Pseudomonas moraviensis*	2.743067278	1.10327715	0.094811071	0.136678163	1.47807083	0.29475489
*Pseudomonas protegens*	0.032109301	0.043054117	0.150782232	4.770376921	0.724556419	0.066775111

*Pseudomonas fluorescens* and *Pseudomonas viridiflava* are pathogens of stored broccoli. The relative abundance of *P. fluorescens* and *P. viridiflava* was 15.4% and 4.97%, respectively, immediately after harvest, and 2.76% and 1.24%, respectively, after 3 days of storage in the dark (control). The relative abundance of *P. fluorescens* and *P. viridiflava* was 12.86% and 6.11%, respectively, immediately after (0 days) the UV-C treatment. After 3 days of storage, the relative abundance of *P. fluorescens* and *Pseudomonas viridiflava* in the UV-C, LED, and UL (UV-C + LED) treatment groups was 13.5% and 5%, 19.8% and 2.4%, and 26.3% and 1.2%, respectively. *Escherichia coli* and *Salmonella enterica* are foodborne bacterial pathogens. The relative abundance of these two pathogens was 0.36% and 0.02%, respectively, when broccoli was harvested, and 1.3% and 0.07% after 3 days of storage in the dark (control). The UV-C treatment had no significant effect on the relative abundance of these two pathogens at 0 days, when it was 0.39% and 0.01%, respectively. The relative abundance of *E. coli* and *S. enterica* was 0.99% and 0.03%, respectively, in the UV-C treatment group after 3 days of storage. The relative abundance of these two taxa after 3 days of storage in the red LED and UL treatment groups was 0.73% and 0.04%, respectively, and 0.59% and 0.05%, respectively. No significant differences in the relative abundance of *E. coli* and *S. enterica* were observed between the three different light treatment groups after 3 days of storage, however, the values in the light treatment groups were lower than they were in the untreated control.

**Figure 4:**
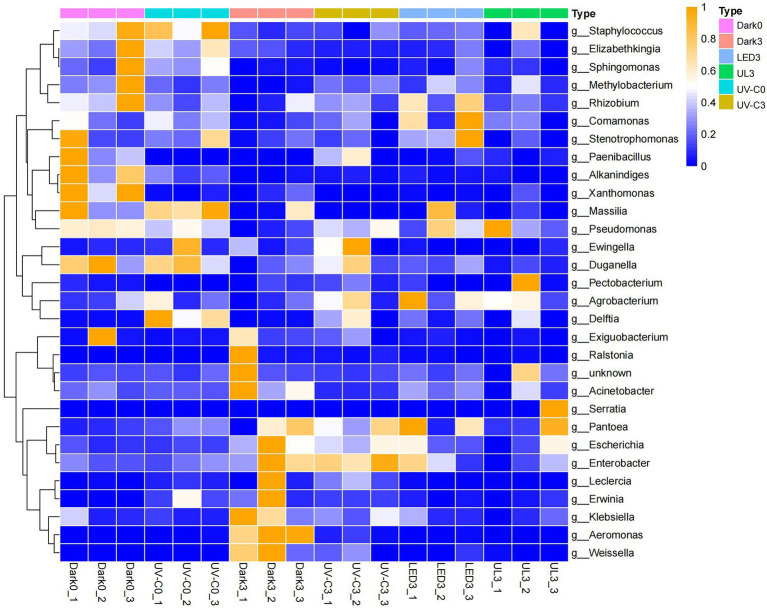
Heat map illustrating the relative abundance of different bacterial genera present in the eluates collected from the surface of broccoli subjected to different light treatments and stored at 20°C for 3 d. Dark0: initial value. The three replicates are Dark0_1, Dark0_2, and Dark0_3. Dark3: broccoli heads were stored in the dark at 20°C for 3 days. The three replicates are Dark3_1, Dark3_2, and Dark3_3. UV-C0: broccoli heads were irradiated with UV-C (3KJ/cm^2^) for 2 min and 29 s then sampled. The three replicates are UV-C0_1, UV-C0_2, and UV-C0_3. UV-C3: broccoli heads were irradiated with UV-C (3KJ/cm^2^) for 2 min and 29 s then stored at 20°C in the dark for 3 days. The three replicates are UV-C3_1, UV-C3_2, and UV-C3_3. LED3: broccoli heads were stored at 20°C under 50 μml/m^2^/s red LED light for 3 days. The three replicates are LED3_1, LED3_2, and LED3_3. UL (UV-C + LED): broccoli heads were irradiated with UV-C (3KJ/cm^2^) for 2 min and 29 s and then stored at 20°C under red LED light (50 μml/m^2^/s) for 3 days. The three replicates are UL3_1, UL3_2, and UL3_3.

A heatmap was generated to examine the changes in the relative abundance of bacteria at the genus level in each treatment group. Results indicated that in control treatment group the relative abundance of *Pantoea*, *Escherichia*, *Enterobacter*, *Erwinia*, *Klebsiella*, *Aeromonas*, and *Weissella* was 5.9%, 0.37%, 2.38%, 0.2%, 0.56%, 0.03%, and 0.03% on day 0. After storage in the dark for 3 days (control), the relative abundance of the same taxa was 15.14%, 1.35%, 7.53%, 2.03%, 1.86%, 3.63%, and 6.64%. In UV-C treatment group (day 0), the relative abundance was 9.62%, 0.39%, 2.64%, 1.31%, 0.29%, 0.05%, and 0.06%, while the same taxa was 16.4%, 1.01%, 8.85%, 0.71%, 0.91%, 0.29%, and 1.63% after storage in the dark for 3 days. The relative abundance of LED and UL treatment groups were 0.16% and 0.83%, 0.74% and 0.59%, 4.77% and 2.03%, 0.33% and 0.39%, 0.54% and 0.33%, 0.07% and 0.02%, and 0.36% and 0.06% (Figure 4).

### Effect of Different Light Treatments on the Fungal Community of Eluates Obtained From the Surface of Stored Broccoli

**Figure 5:**
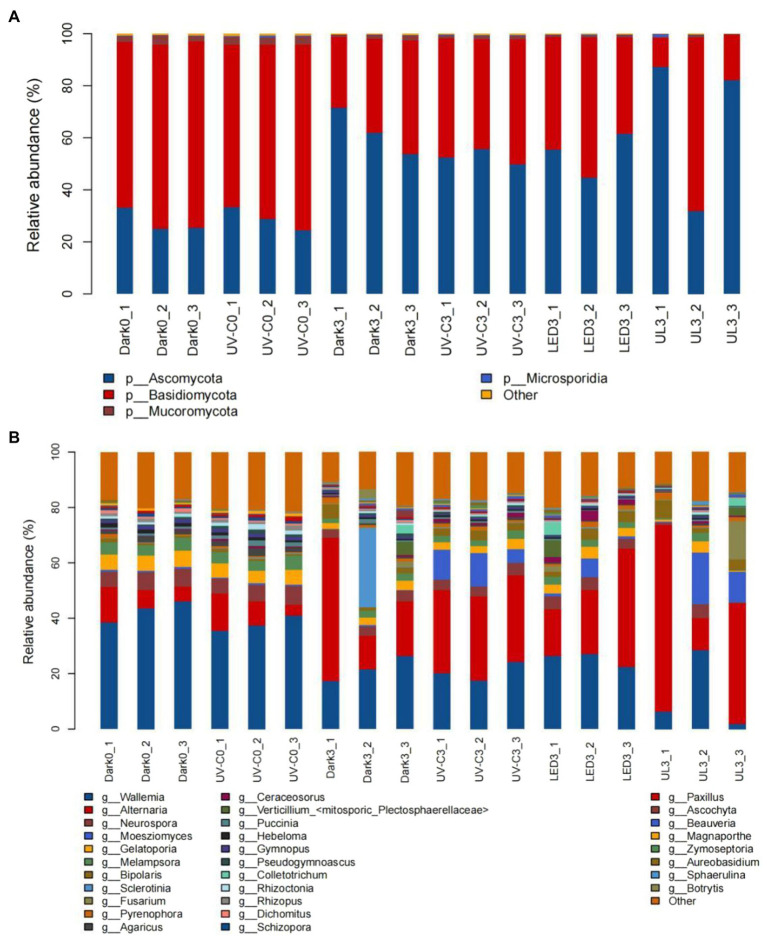
Taxonomic assignments of fungal sequences at the level of phylum **(A)** and genus **(B)** obtained from eluates collected from the surface of broccoli subjected to different light treatments and stored at 20°C for 3 days. Dark0: initial value. The three replicates are Dark0_1, Dark0_2, and Dark0_3. Dark3: broccoli heads were stored in the dark at 20°C for 3 days. The three replicates are Dark3_1, Dark3_2, and Dark3_3. UV-C0: broccoli heads were irradiated with UV-C (3KJ/cm^2^) for 2 min and 29 s then sampled. The three replicates are UV-C0_1, UV-C0_2, and UV-C0_3. UV-C3: broccoli heads were irradiated with UV-C (3KJ/cm^2^) for 2 min and 29 s then stored at 20°C in the dark for 3 days. The three replicates are UV-C3_1, UV-C3_2, and UV-C3_3. LED3: broccoli heads were stored at 20°C under 50 μml/m^2^/s red LED light for 3 days. The three replicates are LED3_1, LED3_2, and LED3_3. UL (UV-C + LED): broccoli heads were irradiated with UV-C (3KJ/cm^2^) for 2 min and 29 s and then stored at 20°C under red LED light (50 μml/m^2^/s) for 3 days. The three replicates are UL3_1, UL3_2, and UL3_3.

The relative abundance of epiphytic fungal taxa in eluates collected from broccoli in the different light treatment groups is presented in Figure 5. A total of 10 Phyla, 76 orders, 169 families, and 298 genera were identified in the collective samples. *Ascomycota* (24.6%–87.2%) and *Basidiomycota* (11.2%–71.7%) were the dominant fungal phyla (Figure 5A). *Wallemia* (1.8%–46.1%), *Alternaria* (3.8%–67.4%), *Neurospora* (0.4%–6.8%), and *Gelatoporia* (0.2%–5.9%) were the predominant identified genera (Figure 5B).

Table 2 lists the relative abundance of the top 20 fungal species found in the broccoli eluates. The different light treatments reduced the relative abundance of some fungi, such as *Sclerotinia sclerotiorum*, *Hebeloma cylindrosporum*, *Gymnopus luxurians*, *Puccinia striiformis*, *Rhizopus delemar*, *Dichomitus squalens*, and *Schizopora paradoxa*. However, the different light treatments also significantly increased the relative abundance of some fungi.

**Table 2:** The relative abundance of the top 20 epiphytic fungal species obtained from the surface of broccoli heads stored at 20°C for 3 days.

	Dark0	UV-C0	Dark3	UV-C3	LED3	UL3
*Wallemia ichthyophaga*	42.67209624	37.85747475	21.69303261	20.59122963	25.33283864	12.17302926
*Alternaria alternata*	8.285083754	8.770225291	27.87174351	30.56584723	27.5093238	40.8264008
*Neurospora tetrasperma*	6.079898603	5.992066186	3.358789561	3.91934092	4.242635842	2.042429339
*Gelatoporia subvermispora*	5.653452186	5.027565264	2.742402079	3.00457033	3.543743593	1.703562186
*Moesziomyces antarcticus*	0.578398735	0.428146959	0.225258552	8.160850128	2.54644622	8.321313435
*Melampsora larici-populina*	4.245122438	4.222800083	2.299205001	2.412661019	2.393518928	1.288235879
*Bipolaris maydis*	0.659660652	0.699613517	2.056892937	2.153300047	2.292300983	2.785848913
*Sclerotinia sclerotiorum*	0.062096425	0.034794711	8.944186615	0.037380136	0.086436099	0.012277471
*Agaricus bisporus*	1.607685883	2.145663009	0.60679342	0.658000612	0.398929133	0.321792793
*Pyrenophora teres*	0.534667588	0.41173783	1.153600226	0.873169669	1.222834028	1.370260155
*Ceraceosorus bombacis*	0.283544787	0.5815507	0.240627537	1.198707635	2.379152514	0.494502302
*Hebeloma cylindrosporum*	1.446733519	0.91187976	0.644106954	0.521967923	0.588718643	0.295807282
*Gymnopus luxurians*	1.141443412	1.131804044	0.668179516	0.654757455	0.530246482	0.214489163
*Puccinia striiformis*	0.717416144	1.41202169	0.678369035	0.58914375	0.275015462	0.30384328
*Rhizoctonia solani*	0.882274644	1.422852811	0.437008258	0.440783004	0.392859813	0.296355506
*Verticillium longisporum*	0.009321402	0.034794711	1.122443652	0.043988542	1.378681707	0.743125932
*Moesziomyces aphidis*	0.072079278	0.049019608	0.034107274	1.154178241	0.317284179	1.641813567
*Rhizopus delemar*	0.95876083	1.230050417	0.406902628	0.246047685	0.159095033	0.081318119
*Dichomitus squalens*	1.052709088	0.601132288	0.455021545	0.3279376	0.347686868	0.246032484
*Schizopora paradoxa*	0.793902331	0.926429553	0.425175552	0.227786181	0.237568911	0.204905905

**Figure 6:**
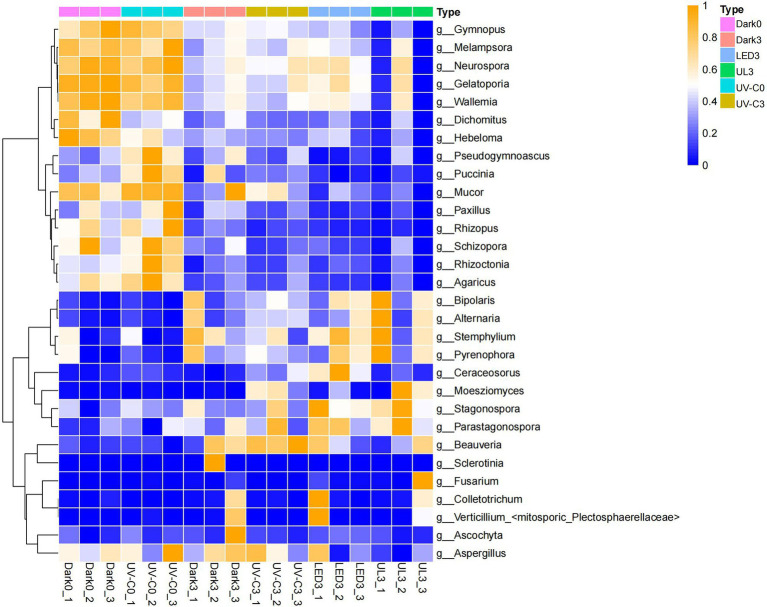
Heat map illustrating the relative abundance of different fungal genera present in eluates collected from the surface of broccoli subjected to different light treatments and stored at 20°C for 3 d. Dark0: initial value. The three replicates are Dark0_1, Dark0_2, and Dark0_3. Dark3: broccoli heads were stored in the dark at 20°C for 3 days. The three replicates are Dark3_1, Dark3_2, and Dark3_3. UV-C0: broccoli heads were irradiated with UV-C (3KJ/cm^2^) for 2 min and 29 s then sampled. The three replicates are UV-C0_1, UV-C0_2, and UV-C0_3. UV-C3: broccoli heads were irradiated with UV-C (3KJ/cm^2^) for 2 min and 29 s then stored at 20°C in the dark for 3 days. The three replicates are UV-C3_1, UV-C3_2, and UV-C3_3. LED3: broccoli heads were stored at 20°C under 50 μml/m2/s red LED light for 3 days. The three replicates are LED3_1, LED3_2, and LED3_3. UL (UV-C + LED): broccoli heads were irradiated with UV-C (3KJ/cm^2^) for 2 min and 29 s and then stored at 20°C under red LED light (50 μml/m^2^/s) for 3 days. The three replicates are UL3_1, UL3_2, and UL3_3.

The constructed heat map presented in Figure 6 reveals that the relative abundance of fungi in the control group was significantly different from the relative abundance in the light treatment groups. The species and species abundance in the control and UV-C treatment groups on day 0 were similar. Fungal pathogens were identified in the different samples. The relative abundance of *Alternaria alternata* was 8.29% when broccoli was just harvested, and 8.77% immediately after the UV-C treatment (day 0). The relative abundance of *A. alternata* was 27.87% after storage in the dark for 3 days (control), while it was 30.57%, 27.5%, and 40.83% in the UV-C, LED, and UL treatment groups, respectively, after 3 days of storage, indicating that the different light treatments had little effect on the growth of *A. alternata*. The relative abundance of *Fusarium avenaceum* after harvest and 3 days of storage in the control was 0.03% and 0.13%. The relative abundance of *Fusarium avenaceum* was not measurable on day 0 or after 3 days of storage in the UV-C treatment group, indicating that UV-C inhibited the growth of this species. The relative abundance of *F. avenaceum*, however, was 0.15% in the LED treatment group and 0.43% in the UL treatment group after 3 days of storage. These result suggest that the red LED treatment negates the negative effect of UV-C light on this pathogen. The relative abundance of *Botrytis cinerea* was 0 at harvest and 0.12% in the UV-C treatment group on day 0, 1.65% in the control after 3 days of storage in the dark, and 0.08%, 0.11%, and 0.05% after 3 days of storage in the UV-C, LED, and UL treatment groups, respectively. These results indicate that the UL treatment had the greatest inhibitory effect on *B. cinerea*.

### Number of Unigenes Identified in the Eluates Collected From the Surface of Broccoli Subjected to Different Light Treatments and Stored at 20°C for 3 Days.

**Figure 7:**
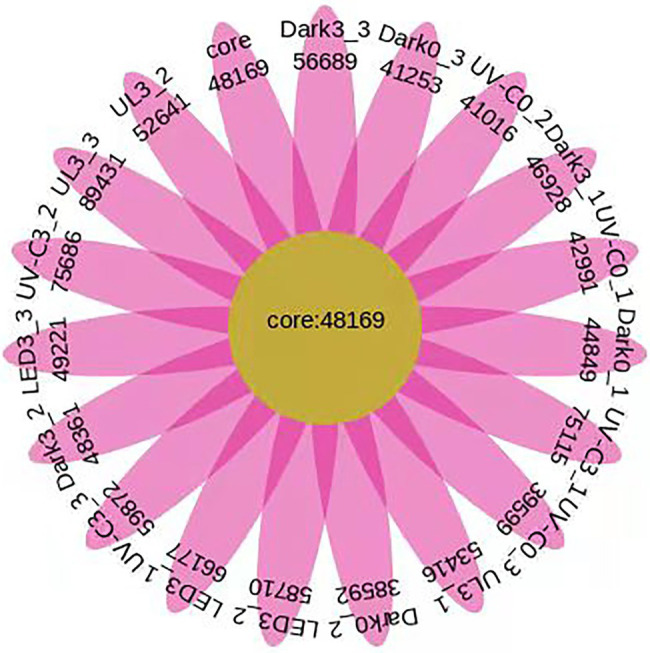
Number of unigenes identified in the eluates collected from the surface of broccoli subjected to different light treatments and stored at 20°C for 3 days. In the Venn diagram, each petal represents a different sample, the middle core region represents the number of genes shared by all samples, and the number of genes unique to a sample is indicated in each petal. Dark0: initial value. The three replicates are Dark0_1, Dark0_2, and Dark0_3. Dark3: broccoli heads were stored in the dark at 20°C for 3 days. The three replicates are Dark3_1, Dark3_2, and Dark3_3. UV-C0: broccoli heads were irradiated with UV-C (3KJ/cm^2^) for 2 min and 29 s then sampled. The three replicates are UV-C0_1, UV-C0_2, and UV-C0_3. UV-C3: broccoli heads were irradiated with UV-C (3KJ/cm^2^) for 2 min and 29 s then stored at 20°C in the dark for 3 days. The three replicates are UV-C3_1, UV-C3_2, and UV-C3_3. LED3: broccoli heads were stored at 20°C under 50 μml/m^2^/s red LED light for 3 days. The three replicates are LED3_1, LED3_2, and LED3_3. UL (UV-C + LED): broccoli heads were irradiated with UV-C (3KJ/cm^2^) for 2 min and 29 s and then stored at 20°C under red LED light (50 μml/m^2^/s) for 3 days. The three replicates are UL3_1, UL3_2, and UL3_3.

A total of 1,028,716 annotated unigenes were identified in the 18 samples of the different light treatments. Furthermore, a total of 124,694, 123,606, 151,978, 174,108, 210,673, and 195,488 genes were identified in the Dark day 0, UV-C day 0, Dark day 3, LED day 3, UV-C day 3, and UL day 3 samples, respectively (Figure 7).

## Discussion

Many vegetables, including broccoli, are eaten raw or minimally processed in salads. Vegetables have a high nutritional value, are beneficial to human health, and are an integral part of a healthy diet (Hanif et al., 2006). The number of foodborne outbreaks related to the consumption of fresh agricultural products has increased over time, estimated at 14.8% in the United States in 1998 and 22.8% in 2007, and several outbreaks of foodborne illness associated with fresh produce have occurred the in the United States and Canada in recent times (Withewoth, 2016; Self et al., 2019). Therefore, it is essential to develop methods that prevent pathogens from contaminating agricultural products (Wadamori et al., 2017). Decay and loss of quality due to pathogenic fungi is also one of the main causes of postharvest losses of fresh fruits and vegetables (Zhu et al., 2018). In our previous study, it was found that the combined treatment of UV-C and red LED irradiation could effectively maintain the sensory quality of broccoli by improving antioxidant enzyme activity, inhibiting the rate of weight loss rate and decreasing the accumulation of malondialdehyde (Liu et al., 2020). In the present study, the microbiota in eluates obtained from the surface of broccoli that were subjected to different light treatments and stored for 3 days at 20°C were analyzed using metagenomic sequencing on an Illumina Hiseq platform.

Results of our study indicated that the microbiota in eluates of broccoli that received different light treatments varied. These differences were associated with different degrees of broccoli decay in the different treatments. After 3 days of storage at 20°C, the untreated, control group had the greatest degree of broccoli decay, indicating that the light treatment had an inhibitory effect on rot development caused by bacteria on the surface of broccoli. The degree of yellowing of broccoli in the different treatments also varied. After 3 days of storage at 20°C, the UL treatment group exhibited the least amount of yellowing and had a good sensory quality (Figure 1). The epiphytic bacterial community of broccoli at the phylum level was predominantly composed of *Proteobacteria* and *Firmicutes*, and the fungal community was predominately composed of *Ascomycota* and *Basidiomycota*. This composition has also been reported in other microbiome studies of strawberry, apples, peaches, peppers, tomatoes, Chinese cabbage, and lettuce (Leff and Fierer, 2013; Abdelfattah et al., 2016; Kim et al., 2018). Species found in the eluates that are pathogenic to broccoli or represent foodborne pathogens species included *P. fluorescen* *P. viridiflava*, *E. coli*, and *S. enterica*. The predominant pathogenic fungal genera identified included *A. alternata*, *F. avenaceum*, and *B. cinerea*.

UV-C irradiation has been demonstrated to be an effective treatment for the surface decontamination of fresh-cut produce (Artés et al., 2009) and has been used to prevent or decrease postharvest disease incidence (Stevens et al., 1996), and improve the postharvest quality of crops (Vicente et al., 2005). The use of UV light offers several advantages to food processors as it does not leave any residue, is easy to use, and is lethal to most microorganisms when used at a sufficient dose (Bintsis et al., 2000).

*E. coli* and *S. enterica* are foodborne pathogens that have been responsible for numerous outbreaks of foodborne illness (Kaper et al., 2004; Franz and van Bruggen, 2008). Results of the present study indicate that UV-C, red LED, and UL (UV-C + red LED) light treatments can reduce the relative abundance of *E. coli* and *S. enterica* in harvested and stored broccoli. Our data indicate that the growth of the two pathogens was clearly inhibited by the light treatments. This may be attributed to the ability of UV-C light to inhibit microbes by preventing DNA replication (Mukhopadhyay et al., 2014). The light treatments, however, had little effect on the relative abundance of *P. viridiflava*, a postharvest decay pathogen of broccoli (Hildebrand, 1989). Bacterial head rot of broccoli is attributed to *P. fluorescens* (Li et al., 2010) and is also a potential human pathogen (Jackson et al., 2015). The relative abundance of *P. fluorescens* decreased significantly in untreated broccoli after storage in the dark for 3 days. In contrast, however, the relative abundance of *P. fluorescens* was significantly higher after 3 days of storage at 20°C in broccoli treated with UV-C, red LED, or UL light. Notably, the red LED and UL treatments included constant exposure to red light during the 3 d of storage, which may have been conducive to the growth of *P. fluorescens*. Angarano et al. (2020) reported that red light irradiation did not promote or decrease biofilm formation. Those findings, however, do not explain the increased growth of *P. fluorescens* in our study in broccoli that received the UV-C light treatment, perhaps suggesting that a higher dose of UV-C light is needed to inhibit this pathogen.

*Fusarium avenaceum* has been identified as a pathogen of broccoli kept in long-term storage at low temperature and controlled atmosphere (Mercier et al., 1991). Our present results indicate that UV-C had an inhibitory effect on the growth and reproduction of *F. avenaceum*, relative to the LED and UL treatments. *Botrytis cinerea*, the causal agent of gray mold, is responsible for significant economic losses in fruits and vegetables due to both pre- and postharvest decay (Fillinger and Elad, 2016). All three of the different light treatments used in the present study had a noticeable inhibitory effect on *B. cinerea* in broccoli stored at 20°C for 3 days. The UV-C light treatment was better than the red LED treatment. The UL treatment, which is a combined UV-C and red LED treatment, was the most inhibitory among the three tested light treatments. This finding is consistent with the previous report by Zhu et al. (2018). In that study, exposure to red LED light *in vitro* increased the antifungal activity of bacteria by enhancing bacterial motility and biofilm formation. Analysis of cell-free supernatants of bacteria treated with red LED light also revealed a higher production of the antifungal lipopeptides iturin and fengycin, relative to untreated bacteria (Yu and Lee, 2013). *Alternaria brassicicola* is the causal agent of plant diseases known by different names, such as *Alternaria* leaf blight, leaf spot, storage rot of vegetables, and black mold, and is capable of infecting cabbage, cauliflower, Chinese cabbage, kale, broccoli, and many other vegetables (Nowicki et al., 2012). The light treatments used in the present study, however, had little effect on the relative abundance of *A. brassicicola*. *A. alternata* is a common contaminant in food and animal feed, and some strains are plant pathogens. Light affects many processes in *A. alternata*. Germination of conidia was reported to be delayed under red and far-red light (Igbalajobi et al., 2019). UV-C irradiation at doses of 2, 4, and 6 KJ/m^2^ significantly reduced the level of rot caused by *A. alternata* in melon. The lesion diameter of infections in melon fruits treated with UV-C was significantly reduced by UV-C irradiation, relative to untreated control fruits (Huang et al., 2015). In contrast, results of our present study indicated that red LED irradiation had little effect on the relative abundance of *A. alternata*, while UV-C and UL irradiation increased the relative abundance of *A. alternata*. Although light treatments in our study increased the relative abundance of most of the epiphytic microorganisms resident on the surface of broccoli, light treatments on disease resistance and the maintenance of postharvest quality have been documented in our previous research (Liu et al., 2020) effect of different light. Light treatments may also enhance the disease resistance of broccoli during storage by maintaining specific physiological and biochemical processes, or by increasing the expression of broccoli resistance genes.

## Conclusion

The present study characterized and quantified the bacterial and fungal communities inhabiting the surface of broccoli heads during storage at 20°C and documented the impact of different light treatments on the composition and structure of the epiphytic microbial community. Results indicated that the structure of the epiphytic microbiota of broccoli varied in response to the different light treatments d. UV-C, red LED, and UL (UV-C + red LED) treatments all influenced the microbial community present on the surface of broccoli stored at 20°C for 3 days. The combined treatment of red LED light + UV-C had the greatest beneficial effect on the preservation of broccoli quality in storage, followed by the red LED light treatment, and the UV-C treatment. All three light treatments had an inhibitory effect on the foodborne bacterial pathogens *E. coli* and *S. enterica*. The UL treatment also reduced the presence of the plant and foodborne pathogen *P. viridiflava*. The UV-C treatment inhibited the growth of *F. avenaceum*. All of the light treatments had an inhibitory effect on *B. cinerea* present on the surface of broccoli stored at 20°C for 3 days. The UL treatment was the most inhibitory, and the UV-C treatment was second. Light treatments prolongs the shelf life of broccoli by maintaining its quality during storage and by inhibiting the growth of foodborne pathogenic bacteria, as well as the growth of fungi causing broccoli decay. Further research is needed to better understand the impact of different microbial communities on the postharvest quality and food safety of broccoli.

## Data Availability Statement

The datasets presented in this study can be found in online repositories. The names of the repository/repositories and accession number(s) can be found at: https://www.ncbi.nlm.nih.gov/, PRJNA678189.

## Author Contributions

QW: conceptualization. JZ: validation. JL: writing - review and editing. SZ: formal analysis and writing - original draft. JC: resources. JX: investigation. SY: validation. XY: review. WG: visualization. LG: validation. All authors contributed to the article and approved the submitted version.

## Funding

This work was supported by China Agriculture Research System of MOF and MARA (CARS-23), Collaborative innovation center of Beijing Academy of Agricultural and Forestry Sciences (KJCX201915), Special innovation ability construction fund of Beijing Academy of Agricultural and Forestry Sciences (KJCX20220418, 20210402, 20200427, and 20210437), the National Natural Science Foundation of China (31772022), the Natural Science Foundation of Beijing (6182016), the National Natural Science Foundation of China (31772022 and 32001763), and the Young Investigator Fund of Beijing Academy of Agricultural and Forestry Sciences (QNJJ202235).

## Conflict of Interest

The authors declare that the research was conducted in the absence of any commercial or financial relationships that could be construed as a potential conflict of interest.

## Publisher’s Note

All claims expressed in this article are solely those of the authors and do not necessarily represent those of their affiliated organizations, or those of the publisher, the editors and the reviewers. Any product that may be evaluated in this article, or claim that may be made by its manufacturer, is not guaranteed or endorsed by the publisher.
